# IL-12α deficiency attenuates pressure overload-induced cardiac inflammation, hypertrophy, dysfunction, and heart failure progression

**DOI:** 10.3389/fimmu.2023.1105664

**Published:** 2023-02-13

**Authors:** Umesh Bhattarai, Xiaochen He, Rui Xu, Xiaoguang Liu, Lihong Pan, Yuxiang Sun, Jian-Xiong Chen, Yingjie Chen

**Affiliations:** ^1^ Department of Physiology and Biophysics, School of Medicine, University of Mississippi Medical Center, Jackson, MS, United States; ^2^ College of Sports and Health, Guangzhou Sport University, Guangzhou, China; ^3^ Department of Nutrition, Texas A&M University, College Station, TX, United States; ^4^ Department of Pharmacology and Toxicology, School of Medicine, University of Mississippi Medical Center, Jackson, MS, United States

**Keywords:** IL-12α, inflammation, heart failure, T cells, macrophages, lung remodeling

## Abstract

IL-12α plays an important role in modulating inflammatory response, fibroblast proliferation and angiogenesis through modulating macrophage polarization or T cell function, but its effect on cardiorespiratory fitness is not clear. Here, we studied the effect of IL-12α on cardiac inflammation, hypertrophy, dysfunction, and lung remodeling in IL-12α gene knockout (KO) mice in response to chronic systolic pressure overload produced by transverse aortic constriction (TAC). Our results showed that IL-12α KO significantly ameliorated TAC-induced left ventricular (LV) failure, as evidenced by a smaller decrease of LV ejection fraction. IL-12α KO also exhibited significantly attenuated TAC-induced increase of LV weight, left atrial weight, lung weight, right ventricular weight, and the ratios of them in comparison to body weight or tibial length. In addition, IL-12α KO showed significantly attenuated TAC-induced LV leukocyte infiltration, fibrosis, cardiomyocyte hypertrophy, and lung inflammation and remodeling (such as lung fibrosis and vessel muscularization). Moreover, IL-12α KO displayed significantly attenuated TAC-induced activation of CD4^+^ T cells and CD8^+^ T cells in the lung. Furthermore, IL-12α KO showed significantly suppressed accumulation and activation of pulmonary macrophages and dendritic cells. Taken together, these findings indicate that inhibition of IL-12α is effective in attenuating systolic overload-induced cardiac inflammation, heart failure development, promoting transition from LV failure to lung remodeling and right ventricular hypertrophy.

## Introduction

Heart failure (HF) or Left Ventricular (LV) failure is a condition in which the left heart is unable to pump out sufficient nutrient and oxygen-rich blood into circulation to meet the body’s needs. HF is a major cause of morbidity and mortality worldwide. Patients with HF are commonly suffered from shortness of breath, diminished exercise capacity and swelling (edema) in legs and feet at night. Chronic HF can also lead to WHO class-2 pulmonary hypertension, lung inflammation and vessel remodeling, right ventricular (RV) hypertrophy and RV failure. The pathological progression from LV failure to RV hypertrophy or RV failure will be stated as HF progression in the rest of the manuscript as previous reports (1–6). Chronic pressure-overload such as hypertension or aortic stenosis is one of the major causes for HF in patients (7), and transverse aortic constriction (TAC) is a commonly used method to cause HF by increasing the pressure-overload in experimental animals. Pressure-loading is an important pathogenic trigger of HF.

Recent studies showed that inflammatory response plays an important role in HF development and HF progression (1, 8–11). Thus, previous studies from us and others have demonstrated that inflammatory regulating factors such as IRF1 (12), IRF4 (13), IRF7 (14), TRAF1 (15), protein kinase R (PKR), and PKR-like ER kinase (PERK), contribute to pressure overload-induced HF development and/or HF progression (16, 17). Previous studies also showed that pro-inflammatory cytokines such as tumor necrosis factor (TNF)-α, interleukin (IL)-1β are upregulated in the cardiac tissue and blood in HF patients and/or experimental animals (18), while inhibition of IL-1β, achieved by blocking antibodies, attenuated major cardiovascular events in patients (19), and HF progression in experimental animals (20). In addition, studies demonstrated that pressure-overload causes various immune cell accumulation in cardiac tissues (3, 8–11, 21), while administration of CTLA4-Ig blocking T cell activation (22), induction of endogenous T regulatory cells, and depletion of CD11c^+^ antigen presenting cells are effective in attenuating HF development and/or progression in experimental animals (21). Furthermore, our previous studies demonstrated that chronic HF causes massive lung inflammation as evidenced by accumulations of macrophages, T cells, and dendritic cells, as well as increased proinflammatory cytokines (1, 8, 20, 23, 24). Moreover, modulating lung or systemic inflammatory responses are effective in regulating HF progression from LV failure to pulmonary hypertension and RV hypertrophy (8, 23, 24).

IL-12α is a subunit of heterodimeric cytokine IL-12 and IL-35. IL-12 (IL-12α/IL-12β) is a cytokine composed of a 35KDa light chain IL-12α and a 40KDa heavy chain IL-12β, while IL-35 (IL-12α/Ebi3) is a cytokine composed of IL-12α and a subunit of Ebi3. The IL-12β subunit in combination with the IL-23p19 monomer leads to the formation of IL-23, another important member of the “IL-12 family”. Despite sharing the IL-12α subunit, IL-12 is a pro-inflammatory heterodimeric cytokine mainly produced by dendritic cells, monocytes and macrophages (25), while IL-35 is an inhibitory heterodimeric cytokine that is mainly produced by regulatory T cells and regulatory B cells (26). IL-12α exerts different immune-regulatory functions determined by its heterodimeric cytokines under the specific disease conditions. For example, one study showed that IL-12α suppresses lymphocyte proliferation and ameliorates autoimmune uveitis in mice by antagonizing pathogenic Th17 responses through induction of IL-10 and IL-35-expressing regulatory B cells (27), while other studies demonstrated that inhibition of IL-12α by genetic deletion significantly attenuated macrophage polarization, T cell derived interferon-γ (IFN-γ) production, and cardiac injury repair after myocardial infarction (28). Importantly, clinical trials have demonstrated that co-inhibition of IL-12/IL-23 signaling was effective in treating ulcerative colitis or Crohn’s disease (29–31), while inhibition of IL-23 signaling alone was not effective in treating ulcerative colitis or Crohn’s disease (32, 33), indicating that IL-12 signaling is essential for the development of ulcerative colitis, a disease showed a significantly high risk of heart attack and HF development (34, 35). However, the role of IL-12α in chronic pressure overload-induced cardiopulmonary inflammation, HF development and HF progression has not been previously studied.

To test the hypothesis that inhibition of IL-12α signaling pathway might be effective in attenuating HF development and progression through attenuating cardiopulmonary inflammatory responses, we studied the role of IL-12α gene knockout (KO) in regulating TAC-induced LV inflammation, hypertrophy, and HF progression in both male and female mice. Our findings indicate that IL-12α inhibition, by IL-12α KO, significantly attenuated systolic overload-induced HF development and progression by modulating cardiopulmonary innate and/or acquired immune responses.

## Materials and methods

### Mice and experimental protocol

IL-12α KO and control wild type (WT) C57/6J mice of both genders were used for TAC or sham surgery. As the female mice generally gain less body weight as compared to the male mice, we used male mice at the age of ~7 to 9 weeks and female mice at the age of ~8 to 10 weeks for TAC or sham surgery. The TAC surgery was performed using a 27G needle to create aortic constriction in male mice as previously described (36). A 28G needle was used for the TAC procedure in female mice, as female mice are generally resistant to TAC-induced HF development as compared to male mice under the same aortic constriction. Following the final cardiac functional test at 8 weeks after TAC or sham surgery, cardiac and pulmonary tissues were collected for further histological, immune-histological, and flow cytometry studies. The mice were housed in a temperature-controlled environment with 12 hours light/dark cycles. This study was approved by the Animal Care and Use Committee at the University of Mississippi Medical Center.

### Echocardiography

Echocardiographic images were obtained using a Visualsonics Vevo 2100 imaging system as previously described (36). The heart rate, ejection fraction, fractional shortening, end-systolic diameter, and end-diastolic diameter were determined using echocardiography.

### Histological staining

Cardiac and pulmonary samples were fixed in 10% formaldehyde, embedded in paraffin blocks, and sections of 10 µm were sliced. Sirius red and Fast green staining (Chondrex, Inc.) was done to quantify fibrosis in the heart and lung tissues. LV cardiomyocyte size was measured by staining the sliced sections with FITC-conjugated wheat germ agglutinin (AF488, Invitrogen). The cross-sectional area of at least 100 LV cardiomyocytes were measured and averaged to get mean cardiomyocyte size. Tissue fibrosis and cardiomyocyte cross-sectional area were quantified by using ImageJ Software. Infiltrating leukocytes and macrophages in the heart and lung tissues were stained with CD45 (R&D systems, AF114) and Mac2 antibody (R&D systems, AF1197), respectively. CD45 and Mac2 were visualized by using a secondary Alexa Flour-555 conjugated antibody. Pulmonary vessels were stained with smooth muscle actin (SMA) (Proteintech, 14395-1-AP) and Alexa Flour-594 conjugated Isolectin B4 (IB4) (Thermo Fisher Scientific, 121413). SMA was visualized by using a secondary Alexa Flour-555 conjugated antibody. Then, the tissue sections were mounted with a mounting media containing 4’, 6’-diamidino-2-phenylindole (DAPI) to visualize the nucleus. All of the histological images were taken using the Mantra Quantitative Pathology Imaging System (Perkin Elmer) and the immuno-histological images were analyzed by inForm software version 2.2.1 (Perkin Elmer) to quantify infiltrating leukocytes and macrophages in the tissues.

### Western blot analysis

LV tissue samples were ground in liquid nitrogen and sonicated in PBS with 1X phosphatase and protease inhibitors. The protein content in the sample was quantified using the BCA protein assay kit (ThermoFisher Scientific, 23225). Then, 40µg protein was loaded on each well and separated using SDS-PAGE and transferred into methanol-activated PVDF membrane. The membrane was then blocked with 5% non-fat milk in TBST for 1 hour. Then, the membrane was incubated with primary antibodies for cardiac β-myosin heavy chain (Abcam, ab50967 at 1:1000 dilution), atrial natriuretic peptide (Thermo Fisher Scientific, PA5-29559 at 1:1000 dilution), and GAPDH (Cell Signaling Technologies Inc., CST 97166 at 1:1000 dilution) overnight at 4°C. Next day, the membrane was washed with TBST three times, 10 minutes each. The membrane was then incubated with HRP-conjugated secondary antibodies (Abcam ab97064, ab97030 at 1:4000 dilution in 5% non-fat milk in TBST) for 1 hour at room temperature. Then, the membrane was washed with TBST three times, 10 minutes each to remove unbound secondary antibodies. The protein band was detected using an iBright FL1500 instrument (Thermo Fisher Scientific) and the bands were quantified using ImageJ analysis software from the National Institutes of Health.

### Cytokine assay

The heart and lung tissues were ground in liquid nitrogen and sonicated in PBS with 1X phosphatase and protease inhibitors. The tissue lysate was centrifuged at 15000 rcf for 15 minutes to get the supernatant. The concentration of cytokines in the supernatant was measured by LEGENDplex flow-based 13-plex mouse inflammation panel (BioLegend, 740446).

### Flow cytometry

Cells were isolated from lung and flow cytometry was performed as previously described (8). Briefly, lung tissue was minced into small pieces and digested in 5 ml collagenase digestion buffer (HBSS without Ca^++^/Mg^++^ (Life Technologies Corporation), 1mg/dL collagenase D (Roche Diagnostics, Germany)) at 37°C for 30 minutes using a cell dissociator (Miltynyi Biotec) and the cells were passed through a 100 µm cell strainer. Then, the RBC was lysed using 2 ml of RBC lysis buffer (Life Technologies Corporation). The remaining cells were counted using an automated cell counter (Bio-Rad Technologies, Inc.). Dead cells were stained with Zombie aqua dye (Biolegend). The cell suspensions were pre-incubated with anti-mouse CD16/32 antibody to prevent non-specific binding of antibodies to FcRγ and then stained with multi-staining antibodies (**Supplementary Table 1**). The samples were subjected to FACS BD LSRII analysis (BD Biosciences). Data were analyzed using FlowJo_V10 (FlowJo, OR) software. Gating strategies are presented in **Supplementary Figures 1 and 2**.

### Statistical analysis

Data were presented as mean ± SEM. A two-way ANOVA followed by a Bonferroni correction *post-hoc* test was used to test the differences between more than 2 groups. All pair-wise p-values are two sided. The null hypothesis was rejected at p<0.05.

## Results

### IL-12α KO ameliorated TAC-induced LV failure in both male and female mice

**Figure 1:**
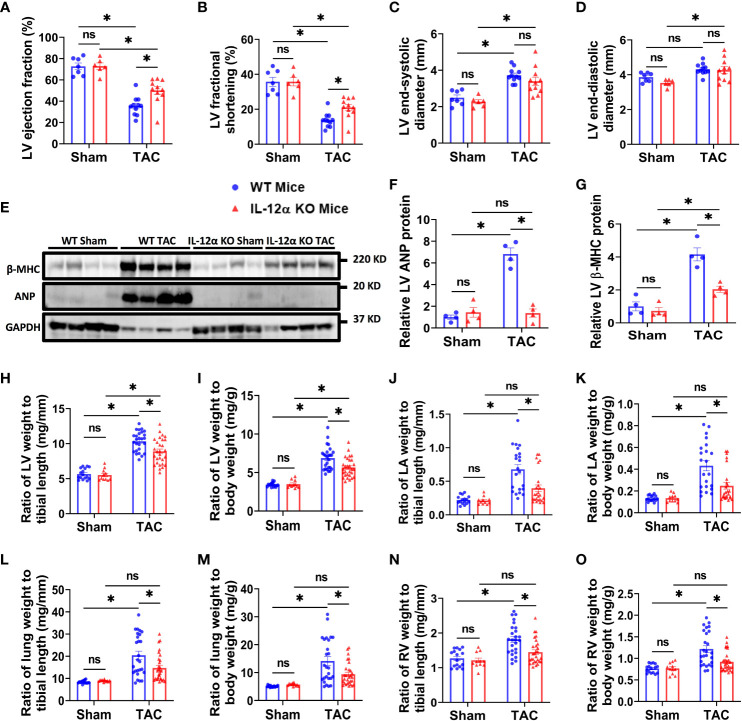
IL-12α KO ameliorated TAC-induced LV failure, LV hypertrophy, increase of lung weight, and RV hypertrophy in male mice. **(A–D)** Quantified data of echocardiographic measurements of LV ejection fraction, LV fractional shortening, LV end-systolic diameter, and LV end-diastolic diameter. **(E–G)** LV β-MHC and ANP expression in corresponding groups. **(H–O)** The ratio of LV weight, LA weight, lung weight, RV weight to tibial length or body weight in corresponding mice. *p<0.05; ns, non-significant.

Since echocardiograms showed that LV ejection fraction and LV fractional shortening were similar in WT and IL-12α KO male and female mice under control conditions (**Figures 1A, B**; **Supplementary Figure 3**). As compared with the corresponding sham group, TAC caused significant decreases of LV ejection fraction and fractional shortening in both WT and IL-12α KO male mice (**Figures 1A, B**). Interestingly, TAC-induced LV dysfunction was significantly attenuated in IL-12α KO male mice as compared with corresponding WT mice (**Figures 1A, B**). TAC-induced increases of LV end-systolic diameter and/or end-diastolic diameter were comparable in WT and IL-12α KO male mice (**Figures 1C, D**). Heart rates were comparable between WT and IL-12α KO male mice in both sham and TAC groups (**Supplementary Table 2**).

Consistent with the observation in male mice, IL-12α KO also significantly attenuated TAC-induced LV dysfunction in female mice as evidenced by significantly less reduction of LV ejection fraction, less reduction in LV fractional shortening, and a smaller increase in the LV end-systolic diameter (**Supplementary Figure 3**).

### IL-12α KO attenuated TAC-induced LV hypertrophy, increase of lung weight and RV hypertrophy in both male and female mice

Western blots demonstrated that TAC-induced expression of LV beta-myosin heavy chain (β-MHC) and atrial natriuretic peptide (ANP), two commonly used biomarkers for pathological cardiac hypertrophy and the consequent heart failure, was significantly attenuated in IL-12α KO male mice (**Figures 1E–G**; **Supplementary Figure 4**).

LV weight, LA weight, lung weight, RV weight, total heart weight, and their ratios to tibial length or body weight were similar in WT and IL-12α KO male and female mice under control conditions (**Figures 1H–O**; **Supplementary Tables 2, 3**, **Supplementary Figure 3**). Body weight and tibial length were comparable in WT and IL-12α KO mice either under control condition or after TAC for both genders (**Supplementary Tables 2, 3**). TAC-induced increases of LV weight, lung weight, and their ratios to tibial length or body weight were significantly attenuated in both IL-12α KO male and female mice as compared with corresponding WT mice of the same gender (**Figures 1H–M**; **Supplementary Tables 2, 3**, **Supplementary Figure 3**). TAC-induced increases of RV weight and its ratios to tibial length or body weight were only significantly attenuated in male IL-12α KO mice as compared with corresponding WT mice (**Figures 1N, O**; **Supplementary Table 2**). These data indicate that IL-12α KO effectively attenuated TAC-induced LV hypertrophy, and the consequent increases of lung weight in mice of both genders.

**Figure 2:**
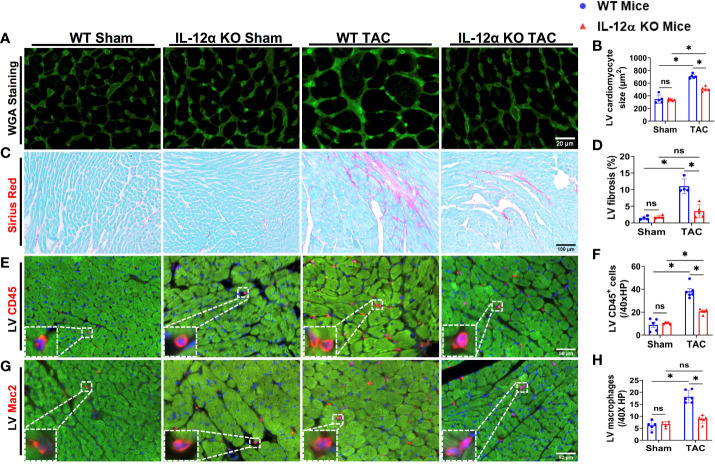
IL-12α KO significantly attenuated TAC-induced LV cardiomyocyte hypertrophy, fibrosis, and leukocyte infiltration. **(A, B)** Representative images and quantified data of LV cardiomyocyte size determined by FITC conjugated wheat germ agglutinin (WGA) staining. **(C, D)** Representative images and quantified data of LV fibrosis performed by Sirius red/Fast green staining. **(E–H)** Representative images and quantified data of LV leukocyte or macrophage accumulation performed by immuno-histological staining. *p<0.05; ns, non-significant.

Since IL-12α KO significantly attenuated TAC-induced LV hypertrophy and dysfunction in both male and female mice, we further determined TAC-induced LV cardiomyocyte hypertrophy using FITC-conjugated wheat germ agglutinin (WGA) staining in male mice. As compared with corresponding WT mice, IL-12α KO significantly attenuated TAC-induced increase in LV cardiomyocyte size (**Figures 2A, B**).

### IL-12α KO significantly attenuated TAC-induced LV fibrosis, leukocyte infiltration, and pro-inflammatory cytokines production

Since IL-12α KO significantly attenuated TAC-induced LV hypertrophy and dysfunction in both male and female mice, we further determined TAC-induced LV fibrosis using Sirius red/Fast green staining in male mice. As compared with corresponding WT mice, IL-12α KO significantly attenuated TAC-induced increase in LV fibrosis (**Figures 2C, D**). In addition, LV leukocyte and macrophage infiltrations were significantly increased in WT mice after TAC, while TAC-induced LV leukocyte and macrophage infiltrations were significantly attenuated in IL-12α KO mice (**Figures 2E–H**).

Monocyte chemoattractant protein 1 (MCP1), also commonly referred to as chemokine ligand 2 (CCL2), exerts an important role in regulating the inflammatory response by enhancing the recruitment of monocytes, memory T cells, and dendritic cells to the sites of injury or infection. We found that TAC caused a significant increase of LV MCP1 protein expression in WT mice but not in IL-12α KO mice (**Supplementary Figure 5**). In addition, TAC caused significant increases of LV IL-1α and IL-27 in WT but not in IL-12α KO mice, while TAC did not affect LV IFNβ and IL-6 in both WT and KO mice (**Supplementary Figure 5**). TAC caused similar decrease of LV IL-17A in WT and KO mice (**Supplementary Figure 5**).

### IL-12α KO significantly attenuated TAC-induced pulmonary inflammation, fibrosis, and vessel remodeling

**Figure 3:**
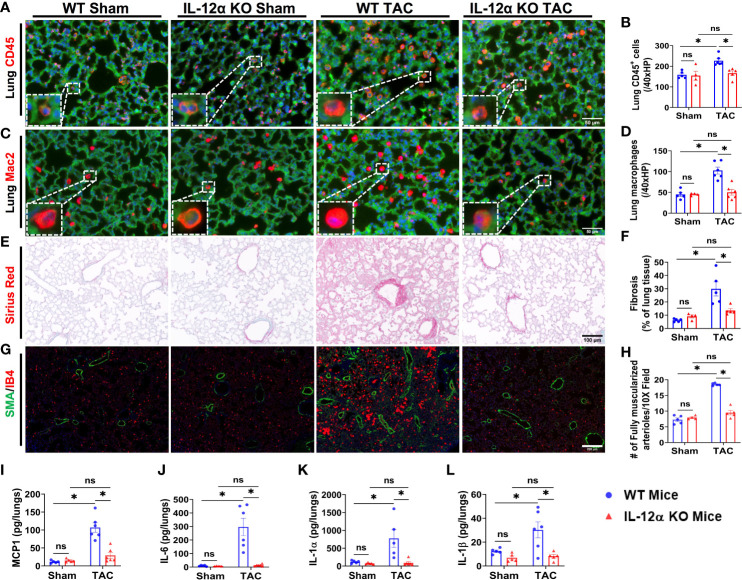
IL-12α KO significantly attenuated TAC-induced pulmonary inflammation, fibrosis, and vessel remodeling. **(A–D)** Representative images and quantified data of infiltrating leukocytes or macrophages in the lung performed by immuno-histological staining. **(E, F)** Representative images and quantified data of lung fibrosis performed by Sirius red/Fast green staining. **(G, H)** Representative images and quantified data of pulmonary vessel remodeling performed by immuno-histological staining. **(I–L)** Alterations of MCP1, IL-6, IL-1α, and IL-1β per lungs sample. *p<0.05; ns, non-significant.

Since our previous studies demonstrated that HF could cause severe lung inflammation and remodeling, and lung inflammation and remodeling could further promote HF progression (24), we also determined lung leukocyte infiltration, fibrosis, and vessel remodeling in male mice. As anticipated, TAC also caused significant accumulation of pulmonary CD45^+^ leukocytes and the macrophage subset in WT mice as compared to control sham mice, while the TAC-induced accumulation of pulmonary CD45^+^ leukocytes and macrophages was significantly attenuated in IL-12α KO mice (**Figures 3A–D**). TAC also caused significant increase of lung fibrosis in WT mice as compared to control sham mice, while TAC-induced lung fibrosis was significantly attenuated in IL-12α KO mice as compared with corresponding WT mice (**Figures 3E, F**). Moreover, the number of muscularized pulmonary vessels were significantly increased in WT mice after TAC, while TAC-induced pulmonary vessel remodeling was significantly attenuated in IL-12α KO mice (**Figures 3G, H**).

Furthermore, pulmonary MCP-1 was also significantly increased in WT mice after TAC, while TAC-induced increase of pulmonary MCP1 was significantly attenuated in IL-12α KO mice (**Figure 3I**). Moreover, the concentrations of pro-inflammatory cytokines such as interleukin-6 (IL-6), interleukin-1α (IL-1α), interleukin-1β (IL-1β), granulocyte macrophage colony-stimulating factor (GM-CSF), interleukin-27 (IL-27), tumor necrosis factor α (TNFα), and interferon β (IFNβ) were significantly increased in WT mice after TAC, while TAC-induced increases in these pro-inflammatory cytokines were abolished in the lung of IL-12α KO mice (**Figures 3J–L**, **Supplementary figure 6**). TAC did not significantly affect lung interleukin-17A (IL-17A) expression in both WT and KO mice (**Supplementary Figure 6**).

### IL-12α KO significantly attenuated TAC-induced lung dendritic cell accumulation and activation

**Figure 4:**
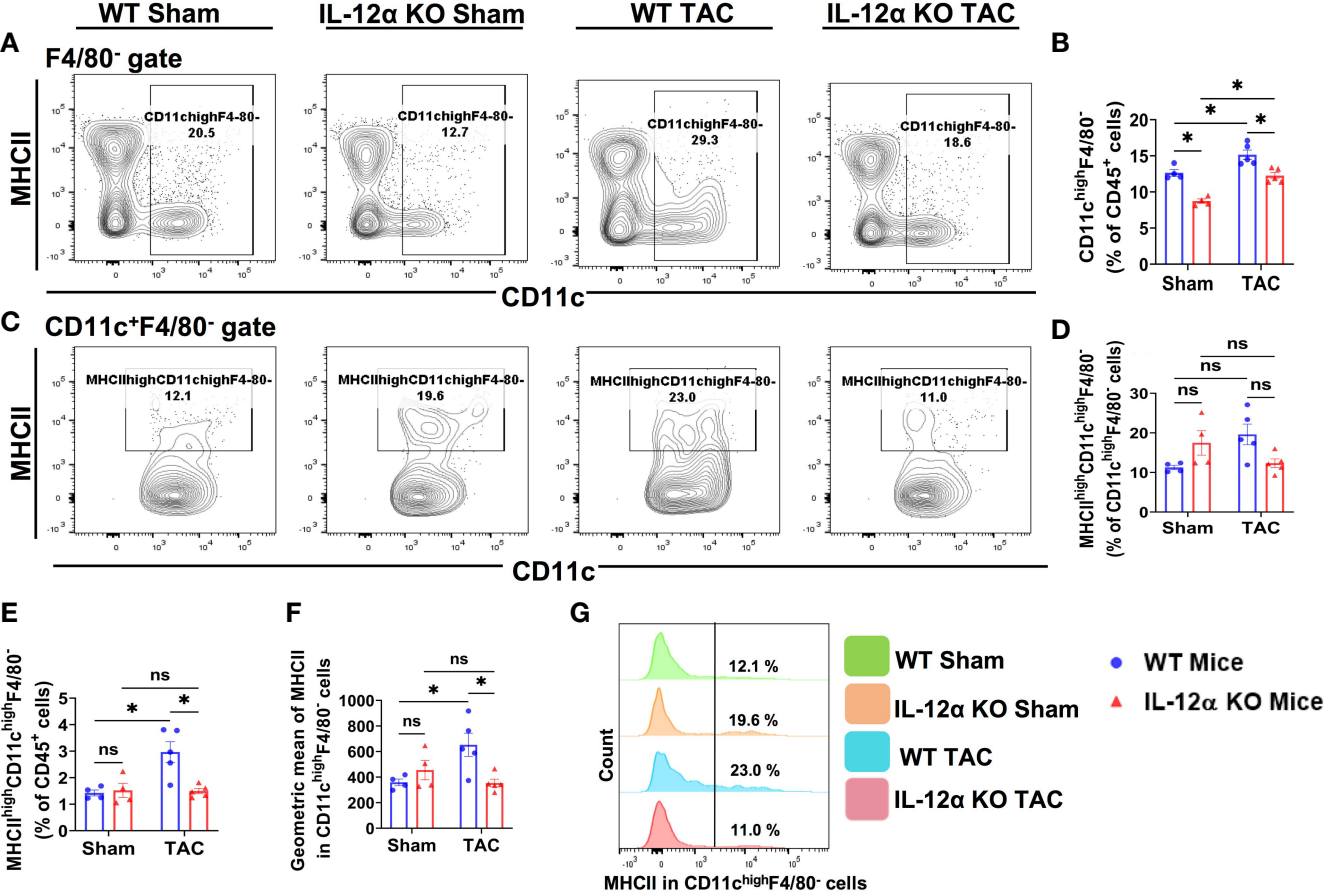
IL-12α KO significantly attenuated TAC-induced lung dendritic cell (CD11c^high^F4/80^-^) accumulation and activation. **(A)** Flow cytometry plots for CD11c^high^F4/80^-^ cells in the lung. **(B)** Percentage of CD11c^high^F4/80^-^ cells within CD45^+^ leukocytes. **(C)** Flow cytometry plots for the identification of MHCII^high^CD11c^high^F4/80^-^ dendritic cells. **(D, E)** Quantified percentage of MHCII^high^CD11c^high^F4/80^-^ dendritic cells within CD11c^high^F4/80^-^ or CD45^+^ leukocytes. **(F)** Mean fluorescent intensity of MHCII in CD11c^high^F4/80^-^ cells. **(G)** Representative histograms showing MHCII expression in CD11c^high^F4/80^-^ cells. *p<0.05, ns, non-significant.

Since IL-12α KO significantly attenuated TAC-induced lung leukocyte accumulation (**Figures 3A, B**), we further determined the alterations of the major lung leukocyte subsets in these mice. As we previously found that CD11c^+^ antigen presenting cells (APCs) play an important role in TAC-induced cardiac hypertrophy and dysfunction (21), we determined the pulmonary dendritic cells in WT and IL-12α KO mice (**Figure 4**). As most lung alveolar macrophages are CD11c^high^ cells, to exclude the influence of alveolar macrophages, lung CD11c^high^F4/80^-^ cells were gated as dendritic cells (**Figure 4A**). We found that the percentages of CD11c^high^F4/80^-^ dendritic cells within CD45^+^ cells were significantly increased in WT mice after TAC, while IL-12α KO significantly attenuated TAC-induced increase of dendritic cells (**Figure 4B**). As increased MHCII expression in dendritic cells generally indicates dendritic cell activation, the percentages of MHCII^high^CD11c^high^F4/80^-^ cells were further determined (**Figures 4C, G**). The percentage of MHCII^high^CD11c^high^F4/80^-^ cells within CD45^+^ cells was significantly increased in WT mice after TAC, while IL-12α KO significantly abolished TAC-induced increase of the percentage of MHCII^high^CD11c^high^F4/80^-^ cells (**Figure 4E**). In addition, the average MHCII expression in CD11c^high^F4/80^-^ cells, as shown by the GEO mean, was significantly increased in WT mice after TAC, while IL-12α KO significantly attenuated the TAC-induced increase of MHCII expression in CD11c^high^F4/80^-^ cells (**Figure 4F**).

### IL-12α KO significantly attenuated TAC-induced pulmonary F4/80^+^ macrophage accumulation and polarization

Lung macrophages exert important roles in regulating the lung inflammatory response. While the alterations of lung macrophages during viral or bacterial infections have been well documented, the alterations of lung macrophage subsets and their activation status after HF development have not been previously described. Thus, we determined lung macrophage subsets and their activation status in WT and IL-12α KO mice after HF development.

**Figure 5:**
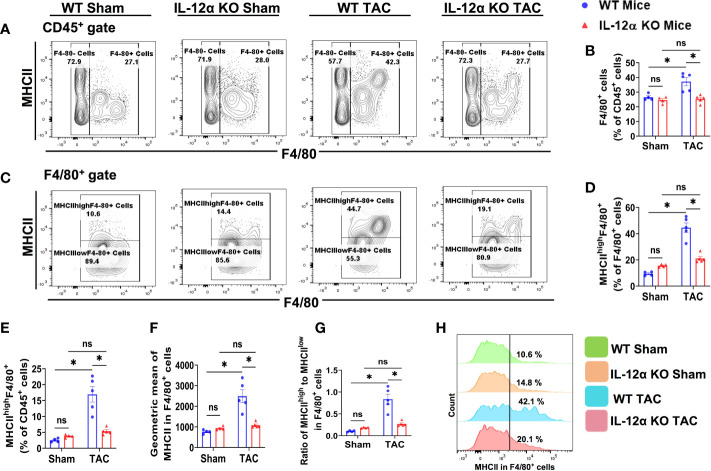
IL-12α KO significantly attenuated TAC-induced pulmonary F4/80^+^ macrophage accumulation and polarization. **(A)** Flow cytometry plots of lung F4/80^+^ macrophages. **(B)** Quantified percentage of F4/80^+^ cells within CD45^+^ cells. **(C)** Flow cytometry plots for detecting MHCII expression in F4/80^+^ cells. **(D, E)** The percentages of MHCII^high^F4/80^+^ cells within F4/80^+^ or CD45^+^ cells. **(F)** Mean fluorescent intensity of MHCII in F4/80^+^ cells. **(G)** Ratio of MHCII^high^ to MHCII^low^ in F4/80^+^ cells. **(H)** Representative histograms of MHCII expression in F4/80^+^ cells. *p<0.05, ns, non-significant.

We found that the percentage of F4/80^+^ macrophages within CD45^+^ cells were also significantly increased in WT mice after TAC, while this increase in the percentage of F4/80^+^ macrophages within CD45^+^ cells was significantly attenuated in IL-12α KO mice after TAC (**Figures 5A, B**). IL-12α KO did not affect the percentage of lung macrophages within CD45^+^ leukocytes under control conditions (**Figures 5A, B**).

Since MHCII expression in macrophages reflects their antigen-presenting capacity and polarization, macrophage MHCII expression and the percentage of MHCII^high^F4/80^+^ cells were determined (**Figures 5C–H**). Interestingly, the percentages of pulmonary MHCII^high^F4/80^+^ macrophages within F4/80^+^ cells or CD45^+^ cells were significantly increased in WT mice after TAC, while IL-12α KO abolished the TAC-induced increase of the percentage of MHCII^high^F4/80^+^ (**Figures 5D, E**). TAC-induced increase of MHCII expression in pulmonary F4/80^+^ cells was also significantly attenuated in the 12α KO mice (**Figure 5F**).

### IL-12α KO ameliorated TAC-induced accumulation of lung alveolar and interstitial macrophages

**Figure 6:**
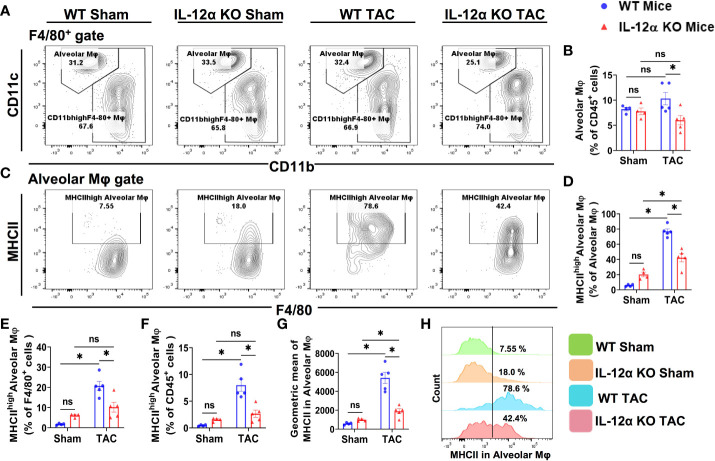
IL-12α KO significantly ameliorated TAC-induced accumulation of lung alveolar macrophages (CD11c^high^CD11b^low^F4/80^+^) and their activation. **(A)** Flow cytometry plots for alveolar macrophages. **(B)** Quantified alveolar macrophages within CD45^+^ leukocytes. **(C)** Flow cytometry plots for the detection of MHCII expression in alveolar macrophages. **(D–F)** Quantified percentages MHCII^high^ alveolar macrophages within alveolar macrophages, F4/80^+^ cells, and CD45^+^ cells, respectively. **(G)** Mean fluorescent intensity of MHCII in alveolar macrophages. **(H)** Representative histograms showing the relative MHCII expression in each group. *p<0.05, ns, non-significant.

**Figure 7:**
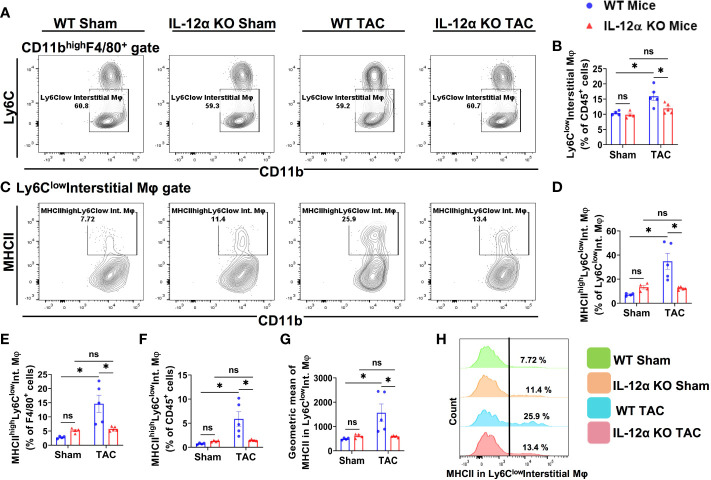
IL-12α KO ameliorated TAC-induced accumulation of Ly6C^low^ interstitial macrophages (Ly6c^low^CD11c^low^CD11b^high^F4/80^+^) and their activation. **(A)** Representative gating plots of Ly6C^low^ interstitial macrophages. **(B)** Quantified percentage of Ly6C^low^ interstitial macrophages within CD45^+^ leukocytes. **(C)** Representative plots for MHCII^high^ macrophages within Ly6C^low^ interstitial macrophages. **(D–F)** Percentages of MHCII^high^Ly6C^low^ interstitial macrophages within Ly6C^low^interstitial macrophages, F4/80^+^, and CD45^+^ cells, respectively. **(G)** Mean fluorescent intensity of MHCII in Ly6C^low^ interstitial macrophages. **(H)** Representative histogram showing MHCII expression in Ly6C^low^ interstitial macrophages. *p<0.05, ns, non-significant.

**Figure 8:**
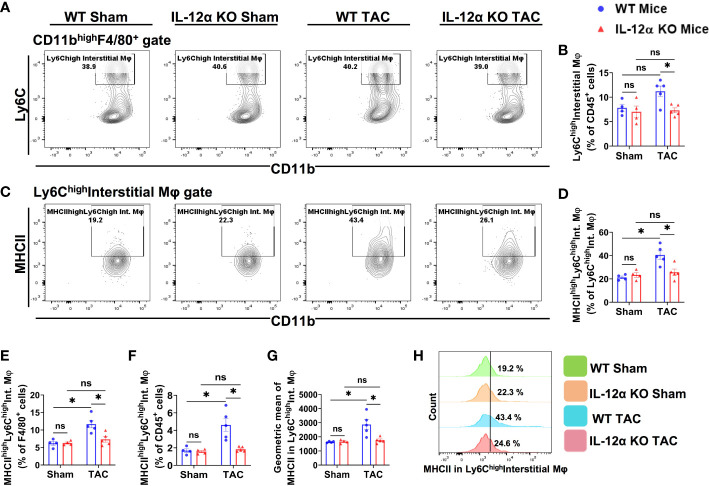
IL-12α KO ameliorated TAC-induced activation of lung Ly6C^high^ interstitial macrophages (Ly6C^high^CD11c^low^CD11b^high^F4/80^+^). **(A)** Representative gating plots for Ly6C^high^ interstitial macrophages. **(B)** Percentages of Ly6C^high^ interstitial macrophages within CD45^+^ cells. **(C)** Representative plots for MHCII^high^ macrophages within Ly6C^high^ interstitial macrophages. **(D–F)** Percentage of MHCII^high^Ly6C^high^ interstitial macrophages within the Ly6C^high^ interstitial macrophages, F4/80^+^, and CD45^+^ cells, respectively. **(G)** Mean fluorescent intensity of MHCII in Ly6C^high^ interstitial macrophages. **(H)** Representative histogram showing MHCII expression Ly6C^high^ interstitial macrophages. *p<0.05, ns, non-significant.

Since lung tissues contain various macrophage subsets, and the composition of lung macrophages contribute to pulmonary inflammation, we further gated the lung F4/80^+^ macrophages according to the expression of CD11c, CD11b, MHCII, and Ly6C, a group of cell surface makers commonly used for the classification of pulmonary macrophage subsets (37–40). Accordingly, F4/80^+^ macrophages were further gated to identify the alveolar macrophages (CD11c^high^CD11b^low^F4/80^+^) (**Figure 6**), Ly6C^low^ interstitial macrophages (Ly6C^low^CD11c^low^CD11b^high^F4/80^+^) (**Figure 7**), and the Ly6C^high^ monocyte derived pro-inflammatory interstitial macrophages (Ly6c^high^CD11c^low^CD11b^high^F4/80^+^) (**Figure 8**), as well as the corresponding subsets according to their MHCII expression.

As shown in **Figure 6**, the percentages of pulmonary alveolar macrophages (CD11c^high^CD11b^low^F4/80^+^) were not significantly increased in either WT or IL-12α KO mice after TAC, but the percentage of pulmonary alveolar macrophages within CD45^+^ leukocytes in IL-12α KO mice was significantly reduced as compared with WT mice after TAC (**Figures 6A, B**). Interestingly, the percentages of MHCII^high^CD11c^high^CD11b^low^F4/80^+^ cells within alveolar macrophages subset, F4/80^+^ cells, or within CD45^+^ leukocytes were all significantly increased in WT mice after TAC, while the above changes were largely abolished in IL-12α KO mice after TAC (**Figures 6C–F, H**). Furthermore, the average MHCII protein expression of pulmonary alveolar macrophages was significantly increased in WT mice after TAC, while IL-12α KO abolished the TAC-induced increase of MHCII protein expression in alveolar macrophages (**Figure 6G**).

As presented in **Figure 7**, TAC caused a significant increase of the percentage of Ly6c^low^ interstitial macrophages within CD45^+^ leukocytes in WT mice after TAC, while IL-12α KO significantly attenuated the TAC-induced increase of lung Ly6c^low^ interstitial macrophages (**Figures 7A, B**). Furthermore, the percentages of MHCII^high^Ly6c^low^ interstitial macrophages within Ly6c^low^ interstitial macrophages subset, F4/80^+^ cells, or within CD45^+^ leukocytes were all significantly increased in WT mice after TAC, while the above changes were completely abolished in IL-12α KO mice after TAC (**Figures 7C–F, H**). In addition, the average MHCII protein expression of Ly6C^low^ interstitial macrophages was significantly increased in WT mice after TAC, while IL-12α KO abolished this TAC-induced increase of MHCII protein expression (**Figure 7G**).

Lung Ly6C^high^CD11c^low^CD11b^high^F4/80^+^ cells are generally recognized as Ly6C^high^ monocyte-derived classical pro-inflammatory interstitial macrophages. As presented in **Figure 8**, we found that TAC did not affect the percentage of Ly6C^high^ interstitial macrophages in both WT and IL-12α KO mice (**Figures 8A, B**). Although it is generally reported that lung Ly6c^high^CD11c^low^CD11b^high^F4/80^+^ macrophages have low MHCII expression or no MHCII expression under basal conditions, while MHCII expression in Ly6c^high^CD11c^low^CD11b^high^F4/80^+^ interstitial macrophages is relatively lower as compared to the MHCII expression in lung alveolar macrophages (CD11c^high^CD11b^low^F4/80^+^) (**Figure 6**), interestingly, MHCII expression in Ly6c^high^ interstitial macrophages was significantly increased in WT mice after TAC (**Figures 8C–H**). IL-12α KO significantly attenuated TAC-induced increase of MHCII expression in Ly6c^high^ interstitial macrophages (**Figures 8C–H**).

### IL-12α KO had no effect on the percentages of pulmonary neutrophils and monocytes in the leukocyte subsets

We also determined the lung neutrophils (CD11b^high^Ly6G^+^), and monocytes (F4/80^-^Ly6C^high^), two important leukocyte subsets in regulating lung inflammatory response. The percentage of neutrophils within CD45^+^ cells was not significantly changed in KO mice under control conditions and in WT and KO mice after TAC (**Supplementary Figure 7**). While TAC did not affect the percentages of lung monocytes within CD45^+^ in both wild type and KO mice, the percentages of lung monocytes were significantly lower in IL-12α KO mice after TAC as compared with WT mice after TAC (**Supplementary Figure 8**).

### IL-12α KO attenuated TAC-induced pulmonary T cell activation

**Figure 9:**
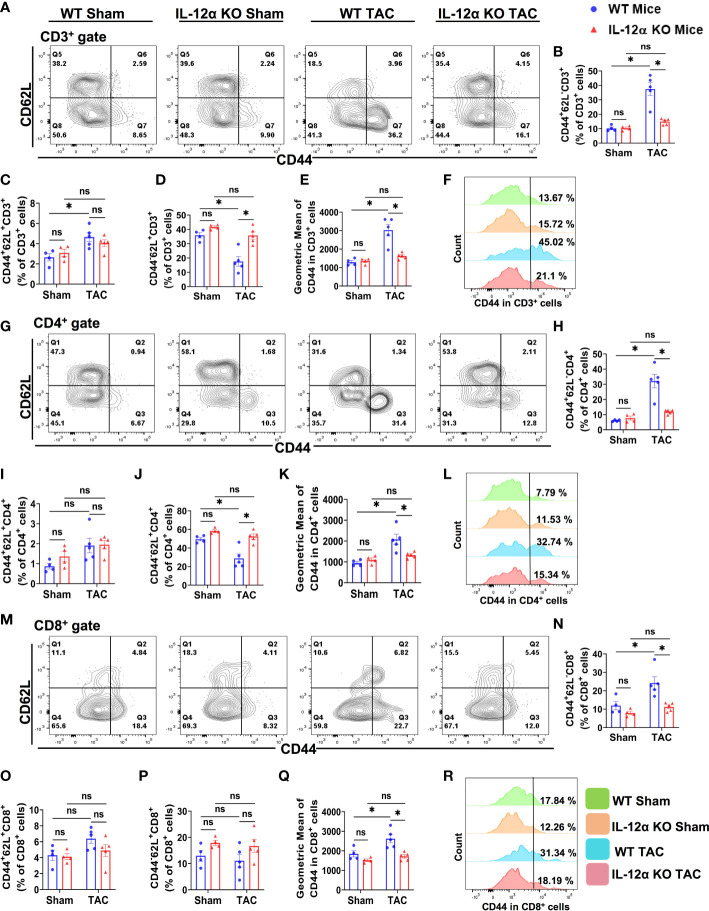
IL-12α KO significantly attenuated TAC-induced T cell activation in the lung. **(A)** Representative plots detecting activation status of CD3^+^ T cells. **(B–D)** Percentages of CD44^+^CD62L^-^ effector memory cells, CD44^+^CD62L^+^ central memory cells, and CD44^-^CD62L^+^ naïve cells within CD3^+^ T cells, respectively. **(E)** Quantified mean fluorescent intensity of CD44 in CD3^+^ T cells. **(F)** Representative histograms of CD44 expression within CD3^+^ T cells. **(G)** Representative plots detecting activation status of CD4^+^ T cells. **(H–J)** The percentages of CD44^+^CD62L^-^ effector memory cells, CD44^+^CD62L^+^ central memory cells, and CD44^-^CD62L^+^ naïve cells within CD4^+^ T cells, respectively. **(K)** Mean fluorescent intensity of CD44 in CD4^+^ T cells. **(L)** Representative histograms of CD44 expression within CD4^+^ T cells. **(M)** Representative plots detecting activation status of CD8^+^ T cells. **(N–P)** The percentage of CD44^+^CD62L^-^ effector memory cells, CD44^+^CD62L^+^ central memory cells, and CD44^-^CD62L^+^ naïve cells within CD8^+^ T cells, respectively. **(Q)** Mean fluorescent intensity of CD44 in CD8^+^ T cells. **(R)** Representative histograms showing CD44 expression within CD8^+^ T cells. *p<0.05, ns, non-significant.

Since our previous studies showed that TAC resulted in increased cardiac and lung T cell activation, and T cell activation exerts an important role in HF development (3), we determined the activation of CD3^+^, CD4^+^, and CD8^+^ T cells in the lung of WT and IL-12α KO mice (**Figure 9**). As anticipated, TAC resulted in significantly increased pulmonary CD3^+^ effector memory T cells (CD44^+^CD62L^-^CD3^+^), increased CD3^+^ central memory T cells (CD44^+^CD62L^+^CD3^+^), and decreased of CD3^+^ naïve T cells (CD44^-^CD62L^+^CD3^+^) cells in WT but not in IL-12α KO mice, and TAC resulted in a significantly smaller increase of pulmonary CD44^+^CD62L^-^CD3^+^ T cells and a smaller decrease of CD44^-^CD62L^+^CD3^+^ T cells in IL-12α KO mice as compared with corresponding WT mice (**Figures 9A–F**). The percentage of CD44^-^CD62L^-^CD3^+^ T cells was significantly decreased in WT mice but not in IL-12α KO mice after TAC (data not shown).

The percentages of effector memory CD4^+^ T cells (CD44^+^CD62L^-^CD4^+^) and effector memory CD8^+^ T cells (CD44^+^CD62L^-^CD8^+^) were also significantly increased in WT after TAC, while the percentage of naïve CD4^+^ T cells (CD44^-^CD62L^+^CD4^+^) were significantly decreased in WT mice after TAC (**Figures 9G, H, J–N, Q, R**). TAC-induced increases of pulmonary effector memory CD44^+^CD62L^-^CD4^+^ and CD44^+^CD62L^-^CD8^+^ T cells were significantly attenuated in IL-12α KO mice (**Figures 9G, H, J–N, Q, R**). The percentages of central memory CD44^+^CD62L^+^CD4^+^ and CD44^+^CD62L^+^CD8^+^ T cells were unaffected by TAC in both WT and IL-12α KO group (**Figures 9G, I, M, O**). The percentages of CD44^-^CD62L^-^CD4^+^ and CD44^-^CD62L^-^CD8^+^ T cells were also unaffected by TAC in both WT and IL-12α KO group (data not shown).

Furthermore, the percentage of lung CD4^+^ within CD3^+^ T cells in the lung was significantly decreased in WT mice after TAC, and this change was abolished in IL-12α KO after TAC (**Supplementary Figure 9**). The percentage of lung CD8^+^ within CD3^+^ T cells was not significantly changed in WT and IL-12α KO mice after TAC (**Supplementary Figure 9**). The percentages of CD3^+^ and CD4^+^ T cells in the CD45^+^ leukocyte population were significantly decreased in WT mice after TAC, while these changes were abolished in IL-12α KO after TAC (**Supplementary Figure 9**). The reduced percentage of CD3^+^ and CD4^+^ T cells within CD45^+^ leukocytes in WT mice after TAC appears to be the outcome of an increased percentage of lung macrophages within CD45^+^ leukocytes in these mice (**Figure 5B**).

## Discussion

In the present study, we investigated the role of IL-12α in regulating LV inflammation, hypertrophy, function, pulmonary inflammation and remodeling, as well as RV hypertrophy in response to chronic TAC in WT and IL-12α KO mice. This study led to several major new findings. First, we found that IL-12α KO significantly attenuated TAC-induced LV inflammation, hypertrophy, fibrosis, and dysfunction. Second, we demonstrated that TAC-induced HF resulted in significant accumulation and activation of lung macrophage subsets, such as alveolar macrophages, Ly6C^low^ interstitial macrophages, and Ly6C^high^ monocyte-derived interstitial macrophages. Third, we found that IL-12α KO significantly attenuated TAC-induced accumulation and activation of lung macrophages. Fourth, we showed that TAC-induced accumulation and activation of pulmonary CD11c^+^ dendritic cells were significantly ameliorated in IL-12α KO mice. Fifth, we demonstrated that IL-12α KO significantly attenuated TAC-induced accumulation and activation of lung CD4^+^ and CD8^+^ T cell subsets in mice. These findings together demonstrate that HF-induced lung remodeling is associated with dramatic changes of several important lung immune cell subsets for both innate and acquired immunity, and inhibition of IL-12α signaling was effective in attenuating pressure overload-induced cardiac inflammation, hypertrophy, dysfunction, lung remodeling, and RV hypertrophy.

One of the major findings is that IL-12α KO significantly attenuated systolic overload-induced LV inflammation, fibrosis, hypertrophy, and dysfunction. The finding of significant cardiac inflammation, fibrosis, hypertrophy, and dysfunction in wild type mice after TAC support the notion that inflammation exerts an important role in HF development. We and others have demonstrated that T cells, T cell activation, CD11c^+^ antigen presenting cells, and macrophages exert important roles in promoting cardiac inflammation, fibrosis, cardiomyocyte hypertrophy and dysfunction (11, 41). The findings that IL-12α KO significantly attenuated TAC-induced cardiac inflammation and failure not only reaffirm the important role of cardiac inflammation in HF development (1, 3, 24), but also demonstrate an important role for IL-12α in cardiac inflammation and HF development. Since IL-12α KO has no detectable detrimental effects on mouse development, cardiac structure and function, blocking IL-12α signaling may be a potentially useful therapeutic approach in treating systolic overload-induced cardiac inflammation and HF development.

Inflammation not only plays an important role in HF development, but also plays an important role in HF progression, a pathological transitional process from LV failure to lung remodeling and consequent RV hypertrophy/failure (1, 3, 24). Our previous studies have demonstrated that severe HF is associated with profound lung inflammation as evidenced by dramatic pulmonary accumulation of macrophages, CD11c^+^ antigen presenting cells, and activated T cells (1, 8–11), while modulating the inflammatory response was effective to control the progression from LV dysfunction to pulmonary remodeling and RV hypertrophy in mice with LV failure (8, 20, 24). For example, we found that HF-induced lung inflammation, fibrosis, and vessel remodeling were significantly attenuated by induction of endogenous Tregs (8), or administration of IL-1β blocking antibodies in mice with existing heart failure (20). Conversely, we found that enhancing lung inflammatory response by air pollution exacerbated the transition from LV failure to lung remodeling and RV hypertrophy in mice with existing LV failure (24). Furthermore, we found that HF is associated with increased lung oxidative stress (23, 24), and reducing oxidative stress by use of an isolevuglandin scavenger effectively attenuated the transition from LV failure to pulmonary inflammation, vessel remodeling and the consequent RV hypertrophy in mice with existing LV failure (23). In the current study, we found that TAC caused significantly increased lung weight, lung fibrosis and leukocyte infiltration, as well as RV hypertrophy in WT mice, while IL-12α KO significantly abolished these TAC-induced changes. Since IL-12α KO significantly attenuated TAC-induced LV failure, and since LV failure can cause lung inflammation, the diminished lung inflammation and remodeling in IL-12α KO mice after TAC is at least in part a reason for less LV failure in these mice. However, since lung inflammation can further directly affect lung remodeling and RV hypertrophy, without affecting LV structure and function (24), and since IL-12α regulated the inflammatory response (42), IL-12α KO might directly suppress lung inflammation and remodeling in HF mice. In the context of the robust lung inflammation as compared with the relatively mild LV inflammation in TAC-induced HF mice (1, 20), the effect of IL-12α on LV dysfunction and its direct effect on lung inflammation might be equally important in promoting lung remodeling. The reduced RV hypertrophy in IL-12α KO mice after TAC might be a collective outcome of reduced lung remodeling and the reduced sensitivity to lung remodeling.

Another interesting finding of the current study is that TAC caused a significant increase of lung alveolar macrophages (CD45^+^/F4/80^+^/CD11c^high^/CD11b^low^ cells), Ly6C^low^ interstitial macrophages (CD45^+^/F4/80^+^/CD11c^low^/CD11b^high^/Ly6c^low^ cells), and Ly6C^high^ monocyte-derived interstitial macrophages (CD45^+^/F4/80^+^/CD11c^low^/CD11b^high^/Ly6c^high^ cells) in WT mice, while the relative MHCII expressions in these macrophage subsets were significantly increased. The dramatically increased lung macrophage subsets after HF, particularly MHCII^high^ cells within these subsets, indicate that these macrophages may not only be useful immune signatures for severe HF, but may also directly regulate the HF-induced lung inflammation and remodeling. The increased MHCII expression in lung macrophages in mice after HF might enhance their antigen presentation capacity and the lung inflammation. The diminished alterations of lung macrophage subsets in IL-12α KO mice after TAC, particularly the reduced MHCII^high^ subsets, indicate that IL-12α might exert an important role in modulating lung macrophage accumulation and polarization during HF progression. Since lung macrophages and dendritic cells regulate T cell activation and IFNγ production through IL-12 (43), the abolished IL-12 production in macrophages and dendritic cells in IL-12α KO mice may be the major mechanism underpinning the reduced T cell activation, and the diminished cardiac and pulmonary inflammation. The pathophysiological roles and the underlying mechanisms of lung macrophages and their subsets in HF progression warrant further investigation.

The present study has several limitations. First, we found that IL-12α KO resulted in decreases of cardiac inflammation, cardiomyocyte hypertrophy, cardiac fibrosis, and lung inflammation. Since each of above changes affects HF development and progression, the current study could not differentiate the relative beneficial effect of inhibition of IL-12α on each of above factors. Nevertheless, the findings from the present study still provide important insights for the detrimental effect of IL-12α on HF development and progression. Second, while both male and female mice were studied in the present study, since IL-12α KO was effective in attenuating TAC-induced cardiac hypertrophy and failure in both male and female mice, we only further characterized the cardiac and pulmonary inflammation and fibrosis in male mice. In the context that gender affects TAC-induced cardiac remodeling (44), there is possibility that female and male mice may have different cardiopulmonary inflammatory responses and remodeling in both WT and IL-12α KO. However, since increased lung weight, lung inflammation and lung fibrosis and RV hypertrophy are commonly observed in both male and female mice after TAC, it is anticipated that inhibition of IL-12α would regulate HF development and progression in both genders through similar molecular pathways.

In summary, we demonstrated that inhibition of IL-12α signaling by IL-12α KO significantly attenuated TAC-induced cardiac and pulmonary inflammation, cardiac dysfunction, and HF progression. IL-12α KO also significantly attenuated TAC-induced pulmonary accumulation of CD11c^+^ dendritic cells, macrophages, and their activation. In addition, we found that IL-12α KO significantly attenuated the overall activation of lung CD3^+^ T cells, and the activation of CD4^+^ and CD8^+^ T cells. Together, these findings clearly indicate that inhibition of IL-12α signaling protected heart against systolic overload-induced cardiac hypertrophy, dysfunction, and the consequent pulmonary remodeling through attenuating cardiac and lung inflammation at least partially through attenuating the activation of infiltrated antigen presenting cells and T cells.

## Data availability statement

The original contributions presented in the study are included in the article/**Supplementary Material**. Further inquiries can be directed to the corresponding author.

## Ethics statement

The animal study was reviewed and approved by Animal Care and Use Committee at the University of Mississippi Medical Center.

## Author contributions

UB, YC, XH, RX, and XL contributed to experimental design, data collection, and data analysis. UB and YC drafted the manuscript. J-XC, LP, and YS edited the manuscript. All authors contributed to the article and approved the submitted version.
