# Supplementary material for: IL-12α deficiency attenuates pressure overload-induced cardiac inflammation, hypertrophy, dysfunction, and heart failure progression
Source: Front Immunol. 2023 Feb 13;14:1105664. doi: 10.3389/fimmu.2023.1105664 (PMC9969090; doi:10.3389/fimmu.2023.1105664)
Supplement: Supplementary file 1 [file DataSheet_1.doc]

**Supplementary Material**

**Supplementary Table 1.** Antibodies used for flow cytometry analysis

| **S.N.** | **Antibody** | **Clone** | **Name of the company** | **Catalog number** |
| --- | --- | --- | --- | --- |
| 1. | BV510-conjugated Live/Dead stain |  | BioLegend | 423101 |
| 2. | anti-mouse CD16/32 | 93 | BioLegend | 101302 |
| 3. | FITC-conjugated anti-CD45 | 30-F11 | Invitrogen | 11-0451-82 |
| 4. | Percp-cy5.5-conjugated anti-CD3 | 145-2C11 | BD Biosciences | 553067 |
| 5. | APC-EF780-conjugated anti-CD4 | GK1.5 | Invitrogen | 47-0041-82 |
| 6. | SB645-conjugated anti-CD8α | 53-6.7 | BD Biosciences | 64-0081-82 |
| 7. | BV711-conjugated anti-CD44 | IM7 | BD Biosciences | 563971 |
| 8. | PE-CY7-conjugated anti-CD62L | MEL-14 | TONBO biosciences | 60-0621-U100 |
| 9. | PE-conjugated anti-TCR γ/δ | UC7-13D5 | BioLegend | 107508 |
| 10. | BV510-conjugated Live/Dead stain |  | BioLegend | 423101 |
| 11. | anti-mouse CD16/32 | 93 | BioLegend | 101302 |
| 12. | AF700-conjugated anti-CD45 | 2D1 | Invitrogen | **56-9459-42** |
| 13. | Percp-cy5.5-conjugated anti-CD11b | M1/70 | BD Biosciences | 550993 |
| 14. | BV711-conjugated anti-MHCII | M5/114.15.2 | BD Biosciences | 563414 |
| 15. | FITC-conjugated anti-F4/80 | BM8 | BioLegend | 123108 |
| 16. | PE-CY7-conjugated anti-CD11c | N418 | BioLegend | 117318 |
| 17. | PE-conjugated anti-Ly6G | 1A8-Ly6g | Invitrogen | 12-9668-82 |
| 18. | BV-605-conjugated anti-Ly6C | HK1.4 | BioLegend | 128035 |

**Supplementary Table 2.** Anatomic data for male wild type and IL-12α knockout mice under Sham condition and after TAC

| **Parameters** | **WT Sham (n=24)** | **IL-12α KO Sham**  **(n=11)** | **WT TAC**  **(n=26)** | **IL-12α KO TAC (n=29)** |
| --- | --- | --- | --- | --- |
| Heart Rate (bpm) | 504.4±9.32 | 496.45±10.07 | 527.85±18.32 | 487.5±12.21 |
| Body Weight (g) | 27.72±0.93 | 27.12±0.87 | 26.61±0.70 | 27.62±0.26 |
| Tibial Length (mm) | 16.89±0.24 | 17.15±0.22 | 17.31±0.10 | 17.50±0.10 |
| LV Weight (mg) | 98.55±4.14 | 94.35±3.92 | 178.68±4.46***** | 158±7.21**#§** |
| LA Weight (mg) | 3.85±0.23 | 3.62±0.27 | 14.50±1.63***** | 9.96±1.84**#** |
| RV Weight (mg) | 21.77±0.89 | 20.72±0.99 | 31.4±1.48***** | 25.32±1.12**#§** |
| RA Weight (mg) | 3.19±0.18 | 3.57±0.25 | 5.48±0.46***** | 4.69±0.27**#** |
| Total Heart Weight (mg) | 126.43±5.02 | 122.25±4.78 | 230.06±7***** | 195.34±8.44**#§** |
| Lung Weight (mg) | 150.41±3.18 | 154.38±2.95 | 352.98±31.02***** | 258.02±20.14**#§** |

Data are mean ± SEM. *p<0.05 compared with WT Sham, #p<0.05 compared with IL-12α KO Sham, §p<0.05 compared with WT TAC.

**Supplementary Table 3.** Anatomic data for female wild type and IL-12α knockout mice under control conditions and after TAC

| **Parameters** | **WT Sham (n=6)** | **IL-12α KO Sham**  **(n=6)** | **WT TAC**  **(n=6)** | **IL-12α KO TAC (n=8)** |
| --- | --- | --- | --- | --- |
| Body Weight (g) | 23.08±0.85 | 23.32±0.37 | 19.78±0.53***** | 20.91±0.42# |
| Tibial Length (mm) | 17.85±0.30 | 17.64±0.12 | 17.27±0.10 | 17.44±0.23 |
| LV Weight (mg) | 82.53±2.76 | 79.08±4.60 | 181.97±4.46***** | 153.52±10.81**#§** |
| LA Weight (mg) | 3.01±0.28 | 2.95±0.19 | 19.88 ±3.60***** | 21.35±6.33**#** |
| RV Weight (mg) | 20.27±1.42 | 17.83±0.75 | 34.28±1.39***** | 28.72±2.54**#** |
| Total Heart Weight (mg) | 108.68±3.80 | 102.62±5.89 | 242.47±10.11***** | 203.6±18.93**#** |
| Lung Weight (mg) | 142.07±2.78 | 149.78±3.85 | 490.93±18.4***** | 323.52±51.66**#§** |

Data are mean ± SEM. *p<0.05 compared with WT Sham, #p<0.05 compared with IL-12α KO Sham, §p<0.05 compared with WT TAC.


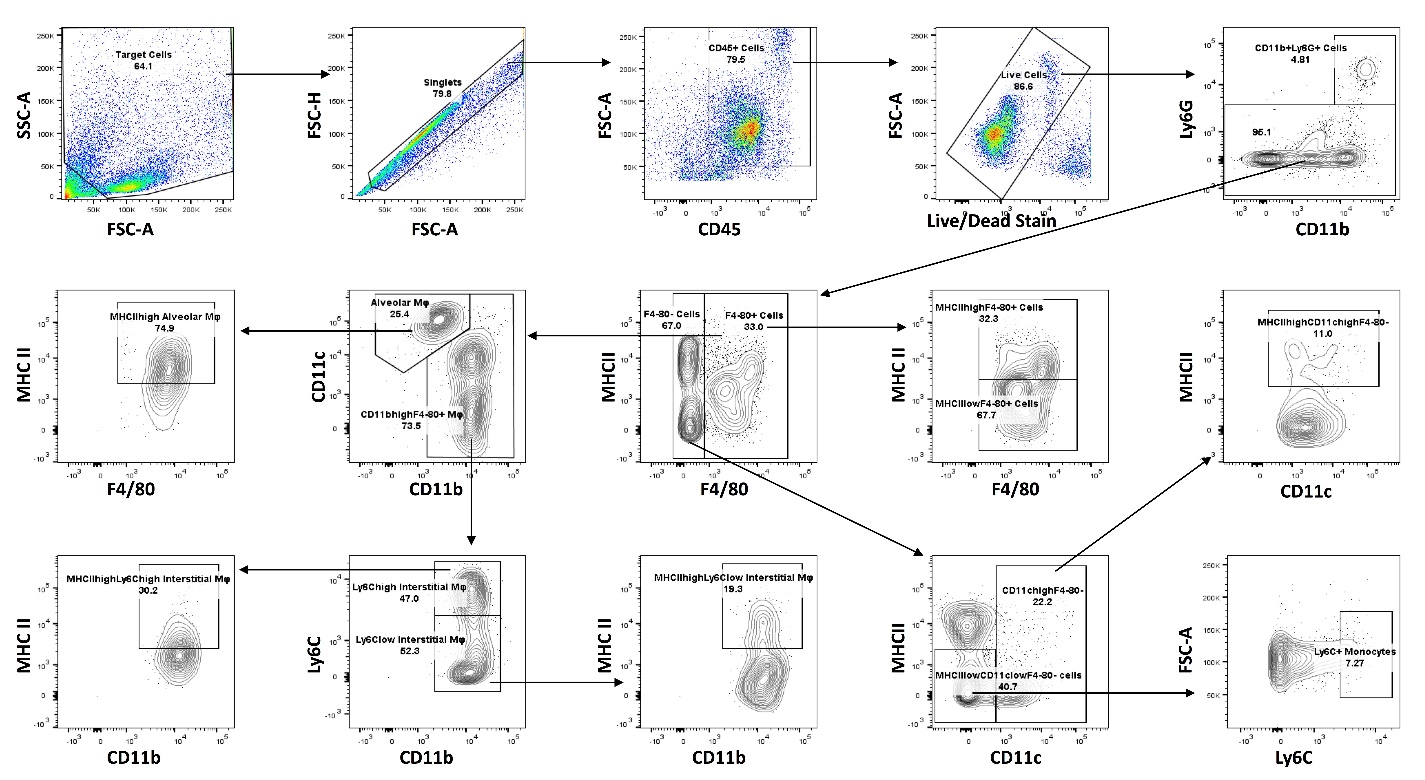


**Supplementary Figure 1.** Gating strategy for the identification of neutrophils, macrophages, and dendritic cells in the lung. For flow cytometry analysis, single cells isolated from the lung were stained with fluorescent conjugated antibodies according to the manufacturer’s instructions and then subjected to FACS BD LSR II analysis.


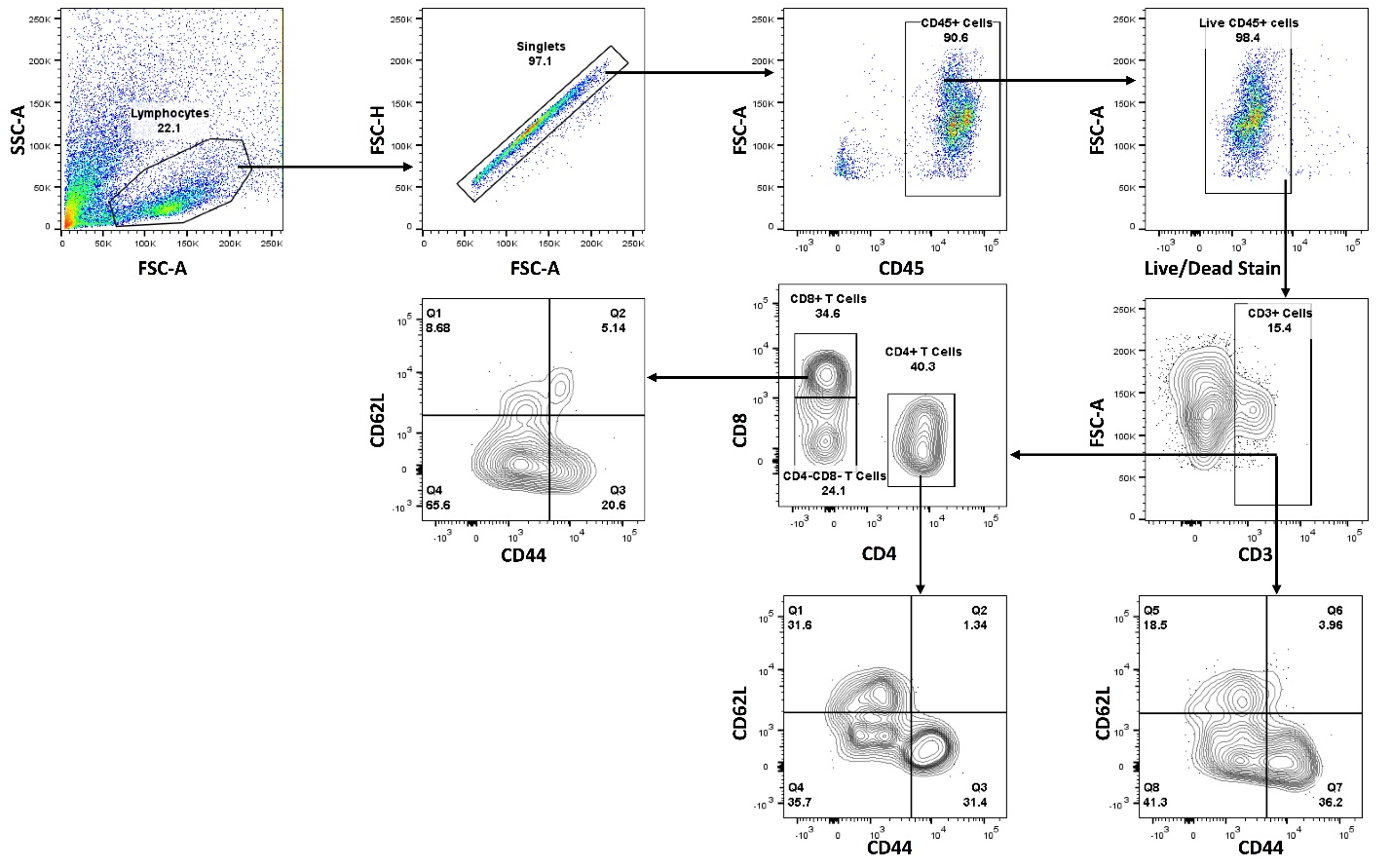


**Supplementary Figure 2.** Gating strategy for the identification and classification of T cells in the lung. For flow cytometry analysis, single cells isolated from the lungs were stained with fluorescent conjugated antibodies according to the manufacturer’s instructions and then subjected to FACS BD LSR II analysis.


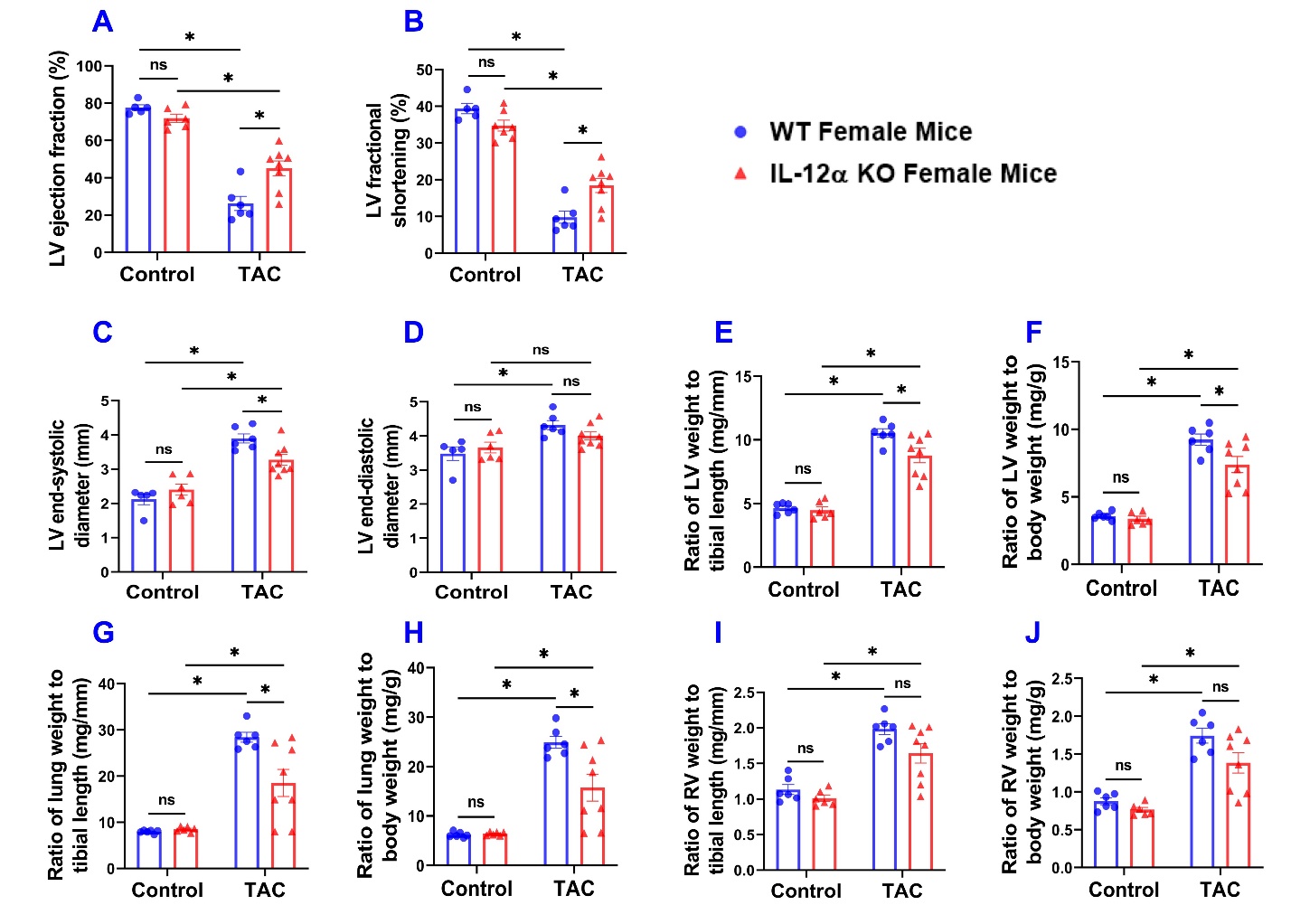


**Supplementary Figure 3.** **(A-D)** Changes in LV function in female WT and IL-12α KO mice under control condition and after TAC. **(E-L)** Changes in ratio of LV weight, lung weight, and RV weight to tibial length or body weight in WT and IL-12α KO female mice under control condition and after TAC. Echocardiographic measurement was done in female WT and IL-12α KO mice 8 weeks after TAC. *p<0.05, ns=non-significant. All values are mean ± SEM.


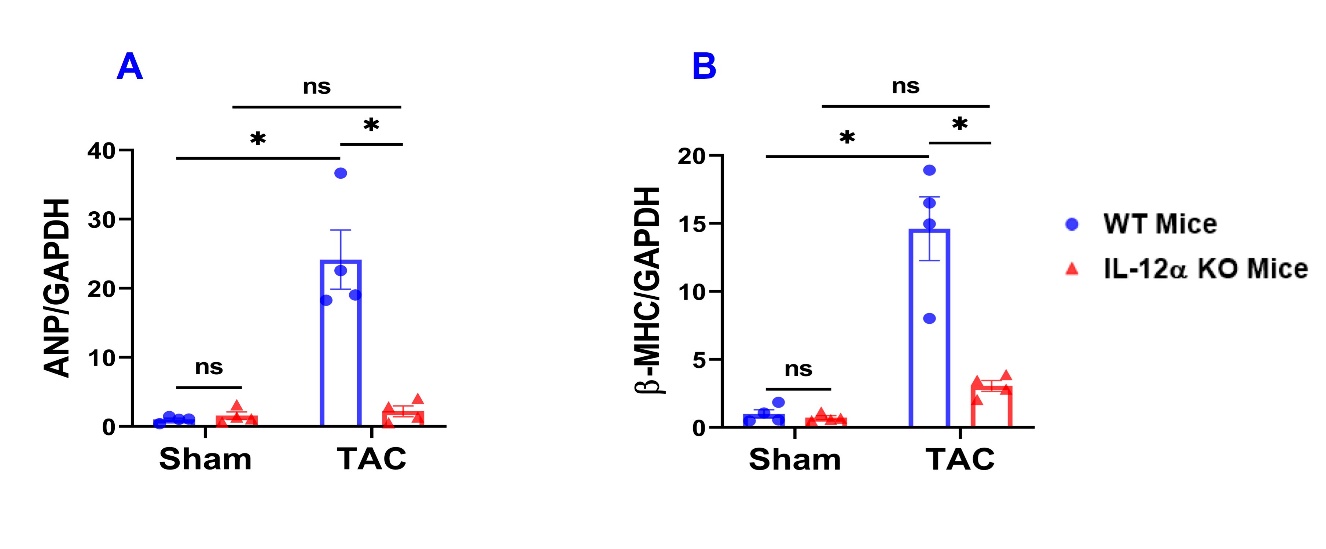


**Supplementary Figure 4.** **(A, B)** Western blot quantification of ratio of atrial natriuretic peptide (ANP) and β-myosin heavy chain (β-MHC) to GAPDH. *p<0.05, ns=non-significant. All values are mean ± SEM.


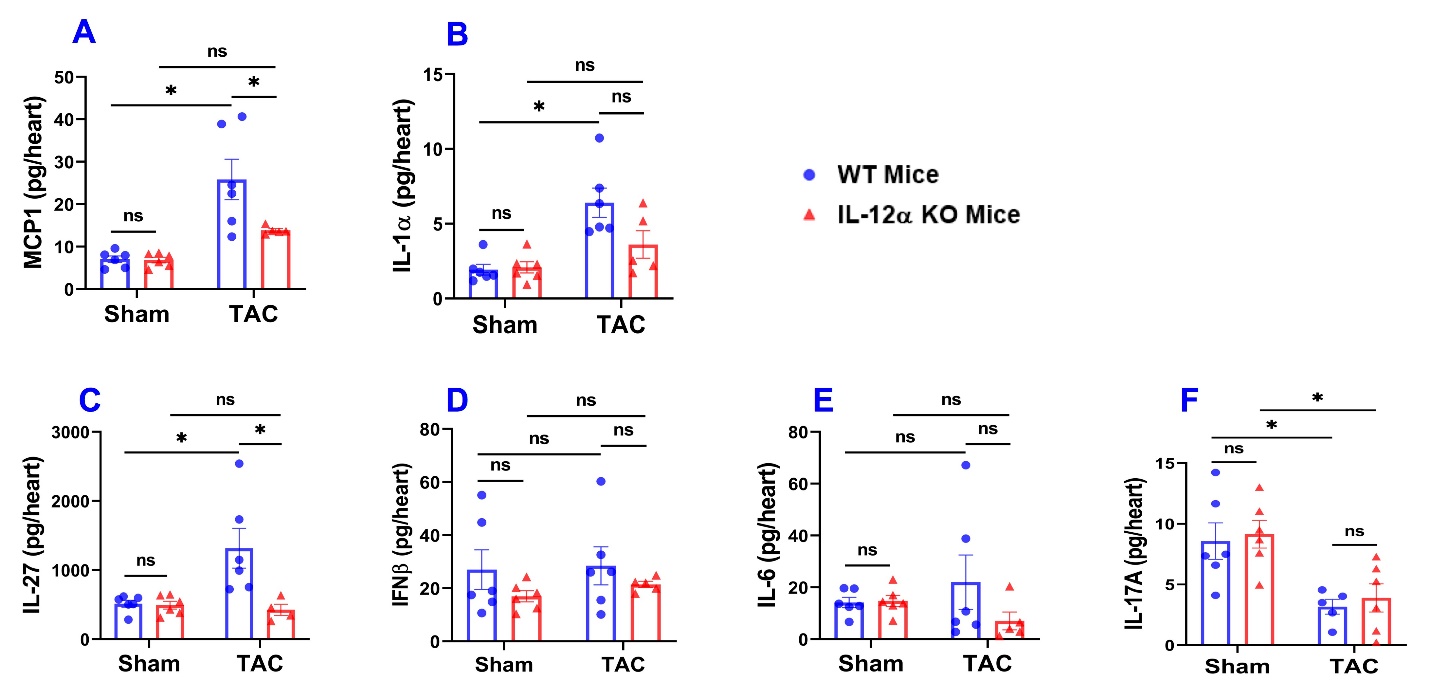


**Supplementary Figure 5. (A-F)** Levels of expression of pro-inflammatory cytokines (MCP1, IL-1α, IL-27, IFNβ, IL-6, and IL-17A) in the heart of WT and IL-12α KO mice under sham and TAC conditions. *p<0.05, ns=non-significant. All values are mean ± SEM.


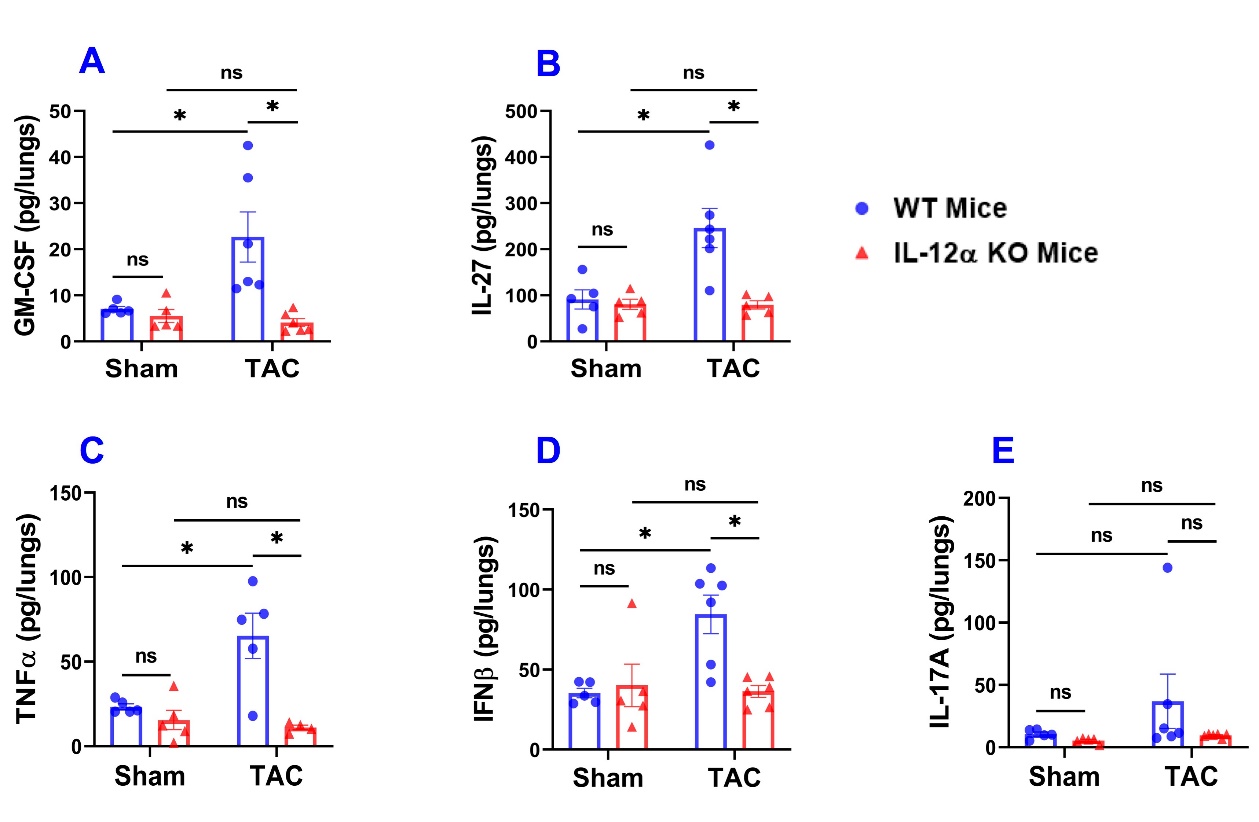


**Supplementary Figure 6. (A-E)** Levels of expression of pro-inflammatory cytokines (GM-CSF, IL-27, TNFα, IFNβ, and IL-17A) in the lungs of WT and IL-12α KO mice under sham and TAC conditions. *p<0.05, ns=non-significant. All values are mean ± SEM.


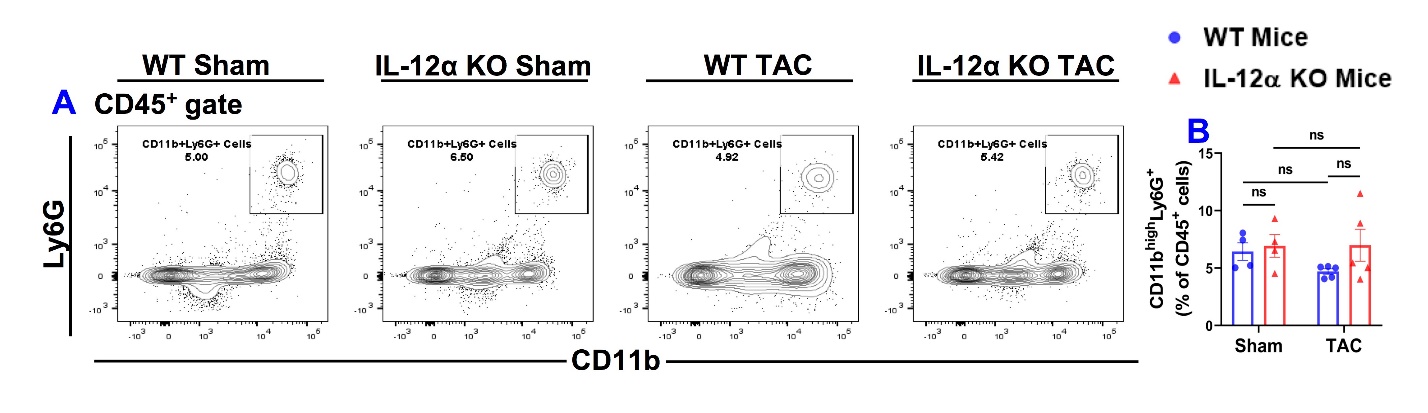


**Supplementary Figure 7.** **(A)** Gating plots for neutrophils (CD11b^high^Ly6G^+^). **(B)** Quantified data of neutrophils within CD45^+^ leukocytes. *p<0.05, ns=non-significant. All values are mean ± SEM.


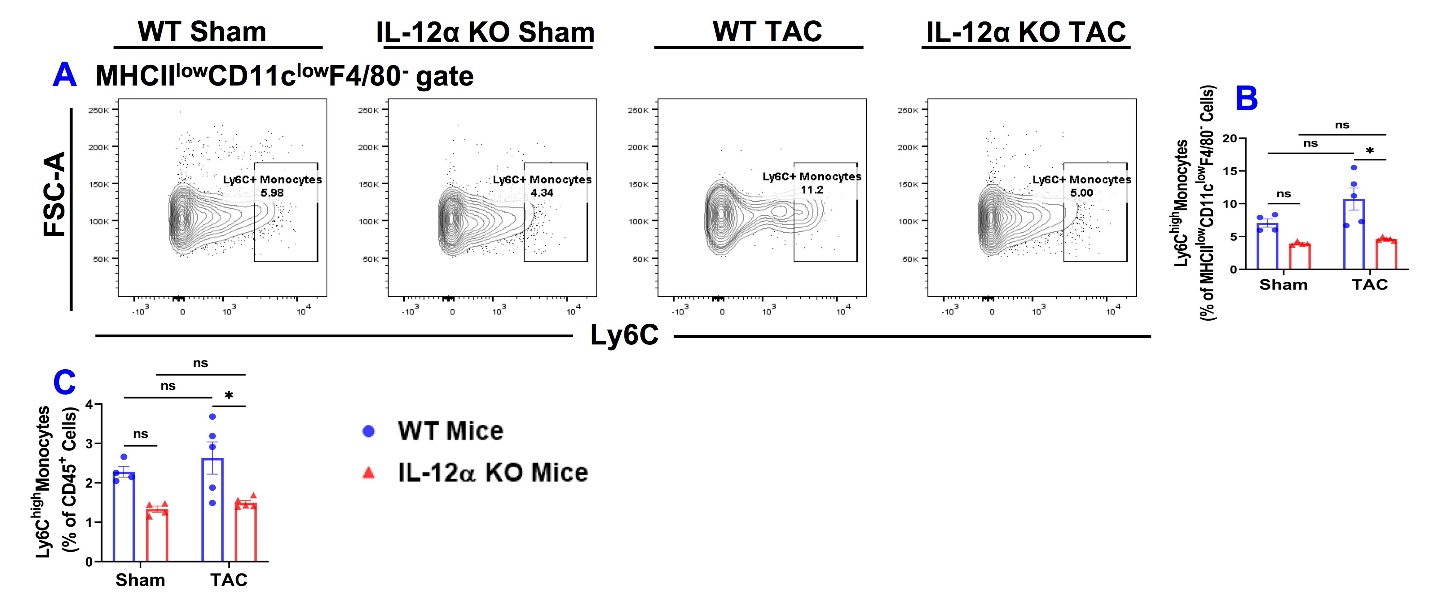


**Supplementary Figure 8.** **(A)** Gating plots for the identification of monocytes (F4/80^-^Ly6C^high^). **(B, C)** Quantified data of monocytes within MHCII^low^CD11c^low^F4/80^-^ cells and CD45^+^ leukocytes, respectively. *p<0.05, ns=non-significant. All values are mean ± SEM.


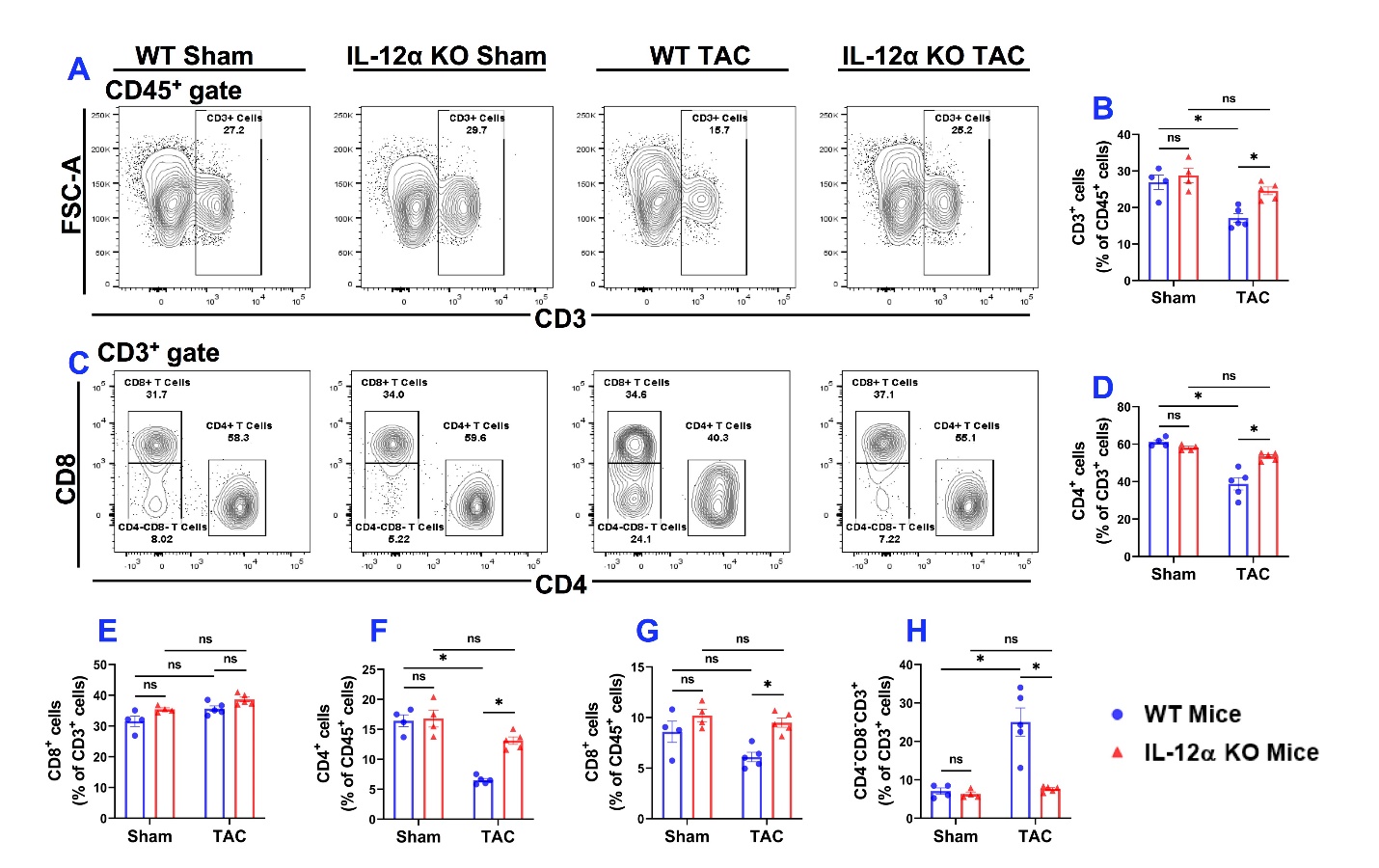


**Supplementary Figure 9.** **(A, B)** Gating plots for the identification of CD3^+^ T cells and their quantification. **(C)** Gating plots for the identification of CD4^+^, CD8^+^, and CD4^-^CD8^-^ T cells. **(D, F)** Quantified data of CD4^+^ T cells within CD3^+^ T cells and CD45^+^ leukocytes, respectively. **(E, G)** Quantified data of CD8^+^ T cells within CD3^+^ T cells and CD45^+^ leukocytes, respectively. **(H)** Quantified data of CD4^-^CD8^-^ T cells within CD3^+^ T cells. *p<0.05, ns=non-significant. All values are mean ± SEM.
